# Emergence of qualitative states in synthetic circuits driven by ultrasensitive growth feedback

**DOI:** 10.1371/journal.pcbi.1010518

**Published:** 2022-09-16

**Authors:** Juan Ramon Melendez-Alvarez, Xiao-Jun Tian

**Affiliations:** School of Biological and Health Systems Engineering, Arizona State University, Tempe, Arizona, United States of America; Duke University, UNITED STATES

## Abstract

The mutual interactions between the synthetic gene circuits and the host growth could cause unexpected outcomes in the dynamical behaviors of the circuits. However, how the steady states and the stabilities of the gene circuits are affected by host cell growth is not fully understood. Here, we developed a mathematical model for nonlinear growth feedback based on published experimental data. The model analysis predicts that growth feedback could significantly change the qualitative states of the system. Bistability could emerge in a circuit without positive feedback, and high-order multistability (three or more steady states) arises in the self-activation and toggle switch circuits. Our results provide insight into the potential effects of ultrasensitive growth feedback on the emergence of qualitative states in synthetic circuits and the corresponding underlying mechanism.

## Author summary

The mutual inhibitory effect between synthetic gene circuits and cell growth produces growth feedback in the host-circuit system. Previous studies have demonstrated that the growth feedback could significantly impact the dynamics of the host-circuit system. However, the complexity of the growth feedback impact is not fully understood. Here, our data analysis displays ultrasensitive growth feedback between the cells and synthetic gene circuits under different growth conditions. To study the effect of ultrasensitive growth feedback on the host-circuit system, we develop a mathematical modeling framework. Our results reveal the emergence of qualitative states on the host-circuit system induced by ultrasensitive growth feedback. We found an emergence of bistability in a simple synthetic gene circuit with a constitutive promoter. Also, tristability could be seen in self-activation and toggle switch circuits. Our research uncovered the effect of ultrasensitive growth feedback in synthetic gene circuits and host interactions. Understanding the effects of ultrasensitive growth feedback could help scientists and engineers identify unexpected outcomes in gene circuits and formulate control strategies.

## Introduction

Synthetic gene circuits are constructed to perform various functions in their host cells, including bacteria, yeast, and mammalian cells [1–5]. The circuit functions heavily depend on the precisely orchestrated dynamical expression of involved exogenous genes. A lot of efforts were used to optimize circuit design and tune the circuit parameters to make them work properly with an assumption that their function is isolated from the host cells [6–8]. However, even when the exogenous circuit does not directly interact with the host cells’ central metabolic pathways, the expression of additional exogenous genes consumes significant levels of cellular resources such as available RNA polymerases and ribosomes that would otherwise be reserved for other cellular functions [9–15]. Thus, the expression of gene circuits inevitably imposes a particular burden on the host cell, changes the physiological state, and reduces the growth rates. In addition, given that host cell growth has a global effect on both endogenous and synthetic genetic circuits [14,16–19], growth feedback universally exists between the host cell physiology and gene circuits and creates a significant amount of uncertainty in predicting circuits’ behavior under real-life cells.

The growth feedback has diverse impacts on the gene circuits [18,20–25]. Under some circumstances, growth feedback can be exploited. For example, Tan et al. showed that a monostable circuit design with non-cooperative self-activation was found to be bistable experimentally, which is driven by growth feedback [20]. On the other hand, growth feedback could impair the circuit functions. Zhang et al. found that the qualitative state of the self-activation circuit could be lost due to the fast dilution of the gene expression by host cell growth [21]. However, in comparison with the self-activation switch, the toggle switch is more robust to growth-mediated dilution. That is, the effects of the growth feedback depend on the gene circuit topologies. Interestingly, Melendez-Alvarez et al. reported unexpected damped oscillatory dynamics of the self-activation circuit by modulating growth feedback with nutrient level [22]. These studies demonstrate how the host growth and circuit interaction can lead to unexpected outcomes depending on the cell condition and gene circuit topologies. However, the influence of growth feedback on gene circuits is not fully understood.

Here, we focused on studying the mechanism of the steady states and the stability changes by nonlinear growth feedback. First, we developed a modeling framework using ordinary differential equations to describe the dynamics of exogenous gene circuits by including growth inhibition and growth-mediated dilution. In addition, a Hill function was used to describe the metabolic burden caused by the exogenous gene expression on growth rate. Parameter estimation based on published experimental data reveals a value of Hill coefficient greater than one, indicating ultrasensitive growth feedback with a high metabolic burden sensitivity. Then, we analyze the mathematical models for several systems to understand the implication of ultrasensitive growth feedback. The analysis suggests that a simple circuit with the constitutive promoter could be bistable with ultrasensitive growth feedback. Further modeling analysis shows that more than two stable steady-states could be seen in self-activation and toggle switch circuits.

## Results

### General modeling framework for synthetic gene circuit with ultrasensitive growth feedback

**Fig 1:**
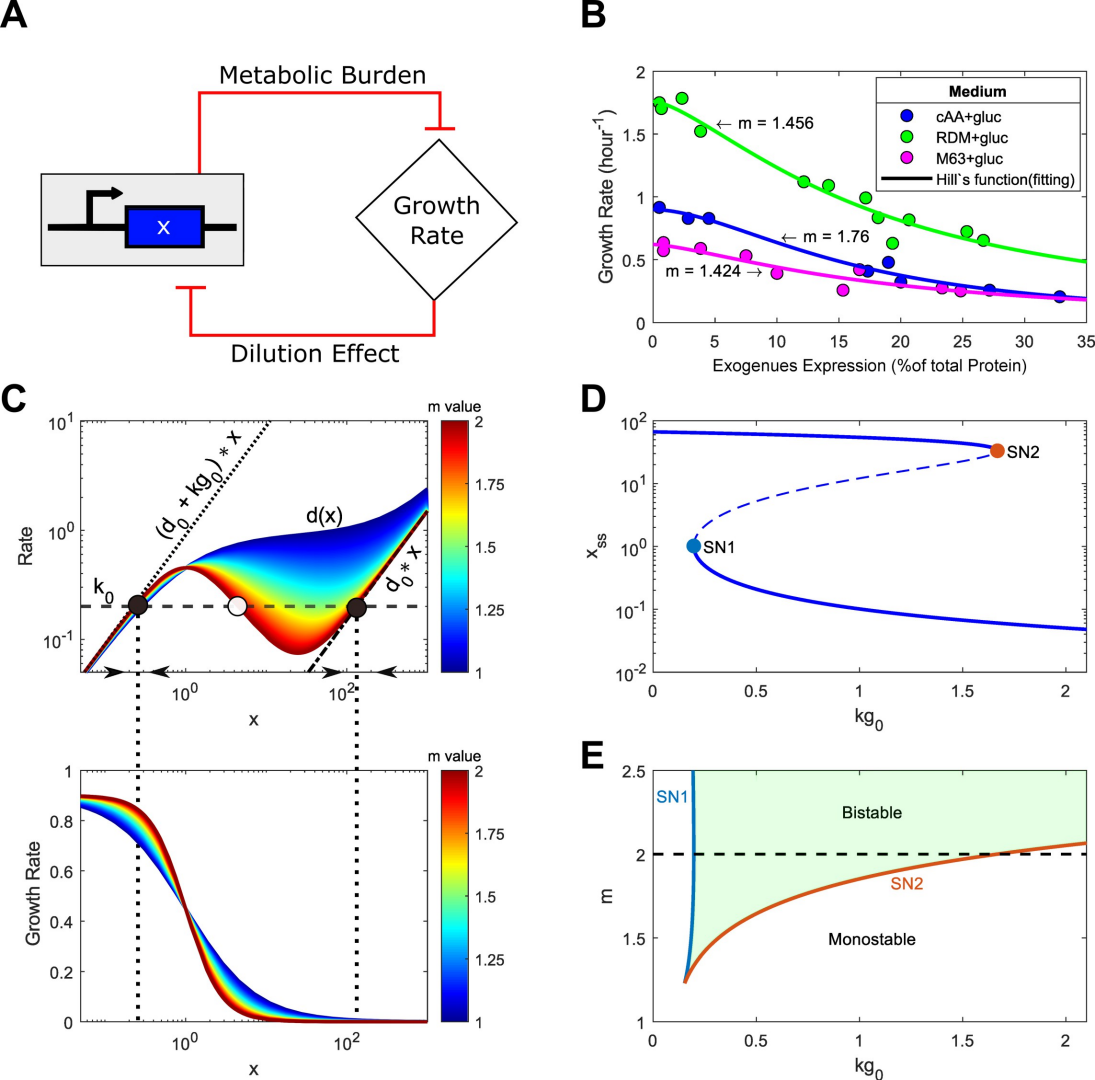
Ultrasensitive cell growth feedback could confer bistability in a simple synthetic gene circuit with a constitutive promoter. (A) Diagram of the mutual inhibition between the host cell growth and synthetic gene expression. (B) Model fitting (solid lines) to experimental data (circle) of the relation between growth rate and exogenous gene expression from different media conditions [14]. (C) Rate balance plot shows the emergence of bistability (top) based on the curves of degradation plus dilution rate versus gene expression (*d*(*x*)) with various *m* values (heatmap) and the curve of production versus gene expression *f*(*x*) = *kg*_0_ (horizontal black dashed line). Dash-dotted black line shows the degradation rate (*d*_0_*x) curve, and the dotted black line shows the degradation plus max dilution rate (*d*_0_*x+kg_0_*x). Solid and open circles represent stable and unstable steady states, respectively. The black arrows on the x-axis denote the directional field. Vertical dotted lines between the top and bottom panels represent the x values at the stable states. The corresponding growth rate curves versus gene expression are shown at the bottom. (D) Bifurcation diagram of the gene expression *x* with respect to *kg*_0_. Solid and dashed lines represent stable and unstable steady states with *m* = 2, respectively. (E) Two-parameter bifurcation diagram with respect to *m* and *kg*_0_. SN1 (blue line) and SN2 (orange line) are the saddle-node bifurcation points shown in (D). The enclosed green region between the saddle nodes represents the bistability. The black dashed line corresponds to the case shown in (D) with *m* = 2.

Exogenous gene expression causes a metabolic burden to the host cell—leading to a reduced cell growth rate, which in turn affects the gene expression through a dilution effect. These mutual inhibitions create a double-negative feedback loop in the host-circuit system (**Fig 1A**). To understand the impacts of growth feedback on the synthetic gene circuits, we followed our previous work model construction [21] and used the following general ordinary differential equations (ODEs) model to describe the dynamics of the gene expression in one gene circuit.

To understand the consequence of this growth feedback on the host-circuit system, we developed a simple mathematical modeling framework,

$$\frac{dx_{i}}{dt}=f_{i}(x)-\overset{Degradation}{\overset{︷}{d_{0,i}*x_{i}}}-\underset{Dilution}{\underset{︸}{GR(x)*x_{i}}},$$

where the *f*(*x*) represents the gene production rate, which could depend on the exogenous gene expression level (*x*) based on the regulations in the circuits, the *d*_0_ is the degradation rate constant of the circuit product *x*_*i*_, and the last term *GR*(*x*)*x represents the dilution rate mediated by host cell growth. It is noted that the growth rate *GR*(*x*) is a function of gene expression level *x*, given that the expression of the synthetic gene circuit causes a metabolic burden that slows down the host cell growth. Previously, *GR*(*x*) was formulated in a Michaelis-Menten format to represent the growth of the cell under a metabolic burden [20,21]. Here, for generality, we use a Hill function:

$$GR(x)=\frac{{kg}_{0}}{{\left(\sum x_{i}/J\right)}^{m}+1},$$

where *kg*_0_ is the maximal growth rate of the host cell without the exogenous genes, *J* indicates the expression capacity for the exogenous gene expression that determines the threshold above which significant metabolic burden is induced. For simplicity, it was also assumed that the metabolic burden caused by exogenous gene expression affects only the growth rate. This framework is suitable for analyzing the system’s steady state in the exponential growth phase. The Hill coefficient indicates the sensitivity of metabolic burden. To quantify the sensitivity of metabolic burden, we used the Hill function to fit experimental data obtained from Ref [14]. The data shows the dependence of the growth rate on exogenous gene expression levels for cells in the exponential growth phase under three different growth conditions (**Fig 1B**). Interestingly, the fitted sensitivity of metabolic burden is greater than one for all the media conditions (**Fig 1B**), suggesting ultrasensitive growth feedback. However, how the sensitivity of the growth feedback affects synthetic gene circuits’ functions is still less understood.

### The emergence of bistability in a simple synthetic gene circuit with a constitutive promoter

To understand the implication of the ultrasensitive growth feedback to the host-circuit system, we first analyzed a simple circuit with a constitutive promoter. For this system, the production rate is expected to be constant and independent of *x*, that is *f*(*x*) = *k*_0_. Applying the general modeling framework gives the following model for this simple system,

$$\frac{dx}{dt}=k_{0}-\underset{d(x)}{\underset{︸}{\left(d_{0}*x+GR(x)*x\right)}}$$

$$GR(x)=\frac{kg_{0}}{{\left(x/J\right)}^{m}+1}$$

First, we performed a rate balance analysis by plotting the production rate (*k*_0_) and dilution plus degradation rate (*d*(*x*)) curves together (**Fig 1C**). The production rate is a constant *k*_0_, indicated as a horizontal curve (**Fig 1C,** black dashed line). Under the condition without cell growth, *d*(*x*) is a linear curve (**Fig 1C**, black dash-dotted line). One stable steady state with high-level gene expression (**Fig 1C**, right solid circle) is found at the intersection point with the production curve. When the growth rate is considered but independent of gene expression, the *d*(*x*) curve is also linear but with a much greater slope (**Fig 1C**, black dotted line). One stable steady-state with low-level gene expression (**Fig 1C**, left solid circle) is found instead. However, when the growth rate depends on the gene expression level, the *d*(*x*) curve is nonlinear and constrained within two dotted and dash-dotted lines (**Fig 1C**). It is worthy to note that the shape of *d*(*x*) curve depends on the values of *m*. **Fig 1C** shows the *d*(*x*) curve (top) and growth rate (bottom) for a range of values of *m*. For *m*>1, the *d*(*x*) curve can exhibit local maximum and minimum, which allow the system to have three steady states at three intersections of the *k*_0_ and *d*(*x*) curves. Two of them (solid circles) are stable, representing the ON state with a high expression but low growth rate and the OFF state with low expression and high growth rate, and the other is unstable (open circle), representing the threshold that separates the two stable states. Intuitively, if circuit gene expression is not above the threshold, the caused burden is not significantly high, thus the cell growth rate is not attenuated much and still leads to a significant dilution that further leads to a decrease of the circuit gene expression and the burden. Under this condition, we can expect that the system stabilizes in one state in which the cell grows fast while circuit gene expression is low. On the other hand, if the burden caused by the circuit is very high, the growth rate is reduced significantly, so the dilution efforts will be significantly reduced, which leads to a further increase in gene expression and metabolic burden. Under this condition, we expect that the system stabilizes in a state in which the cell grows very slow and circuit gene expression is high. This intuitive explanation is indicated by the direction arrows (**Fig 1C**). Therefore, a simple circuit with a constitutive promoter could be bistable with ultrasensitive growth feedback.

One condition for the emergent bistability is that the *d*(*x*) curve is nonmonotonic with both local maximum (*d*(*x*_*max*_)) and minimum (*d*(*x*_*min*_)). Once the *d*(*x*) curve is nonmonotonic, we also need the production rate in the range *d*(*x*_*min*_)<*k*_0_<*d*(*x*_*max*_) so that the three intersection points could be found (See Note A in S1 Text). Given that $d(x)=\left(\frac{{kg}_{0}}{1+{\left(x/J\right)}^{m}}+d0\right)*x$, three parameters, *kg*_0_, *J*, and *m* can change the shape of *d*(*x*) curve. We perturbed these parameters further to understand the underlying mechanism and conditions of emergent bistability. **S1A Fig** shows the difference between the maximum and minimum of *d*(*x*) (Δ*d* = *d*(*x*_*max*_)−*d*(*x*_*min*_)) in the space of *kg*_0_ and *m*, where bistability could be found in the upper-right corner. It is noted that increasing *kg*_0_ and m increase Δ*d*, and thus enhance the range of *k*_0_ for the system to be bistable. That is, an increase of *kg*_0_ or m is beneficial for bistability emergence. The bistability could be lost if *kg*_0_ is reduced (**S1B Fig**, blue to orange curve), and regained with an increase of *m* (yellow curve), and the bistable range could be increased with an increase of *kg*_0_ (purple curve) or further increased with an increase of both *kg*_0_ and *m* (green curve). The boundary curve is $\frac{kg_{0}}{d_{0}}\geq \frac{4m}{{\left(m-1\right)}^{2}}$ (See Note A in S1 Text for detail). Interestingly, this condition is independent of parameter J. Hence, our analysis suggests that a system with relatively high values of *m* and *kg*_0_ sets up the conditions for one steady-state emergence.

To further demonstrate the emergence of bistability in the system due to the growth feedback, we performed a one-parameter bifurcation analysis with respect to *kg*_0_ (**Fig 1D).** Notice that the system is monostable for small and high values of *kg*_0_ corresponding to high and low gene expression states, respectively. While for moderate values of *kg*_0_, the system becomes bistable. For fixed *kg*_0_ in this range, the system shows bistable in a broad range of *k*_0_, as shown in the one-parameter bifurcation diagram (**S1C Fig**). Saddle node points SN1, and SN2 (**Figs 1D** and **S1C**) set the lower and higher values of *kg*_0_ or *k*_0_ for bistability. We further performed a two-parameter bifurcation analysis with respect to *kg*_0_ and *m*, which discloses the change in the *kg*_0_ values of the saddle nodes with respect to the *m* value (**Fig 1E**). The two saddle-node get closer for decreasing values of *m*. The merge of the saddle nodes implies the loss of bistability. Notice that the system can display bistability for values on m between 1 and 2. This is also true in the two-parameter bifurcation analysis with respect to *k*_0_ and *m* (**S1D Fig)**. Remarkably, our analysis suggests that an emergence of a steady-state could occur with *m* values estimated with experimental data. Therefore, bistability emerges with ultrasensitive growth feedback.

### Tristability emergence in the self-activation switch under ultrasensitive growth feedback

**Fig 2:**
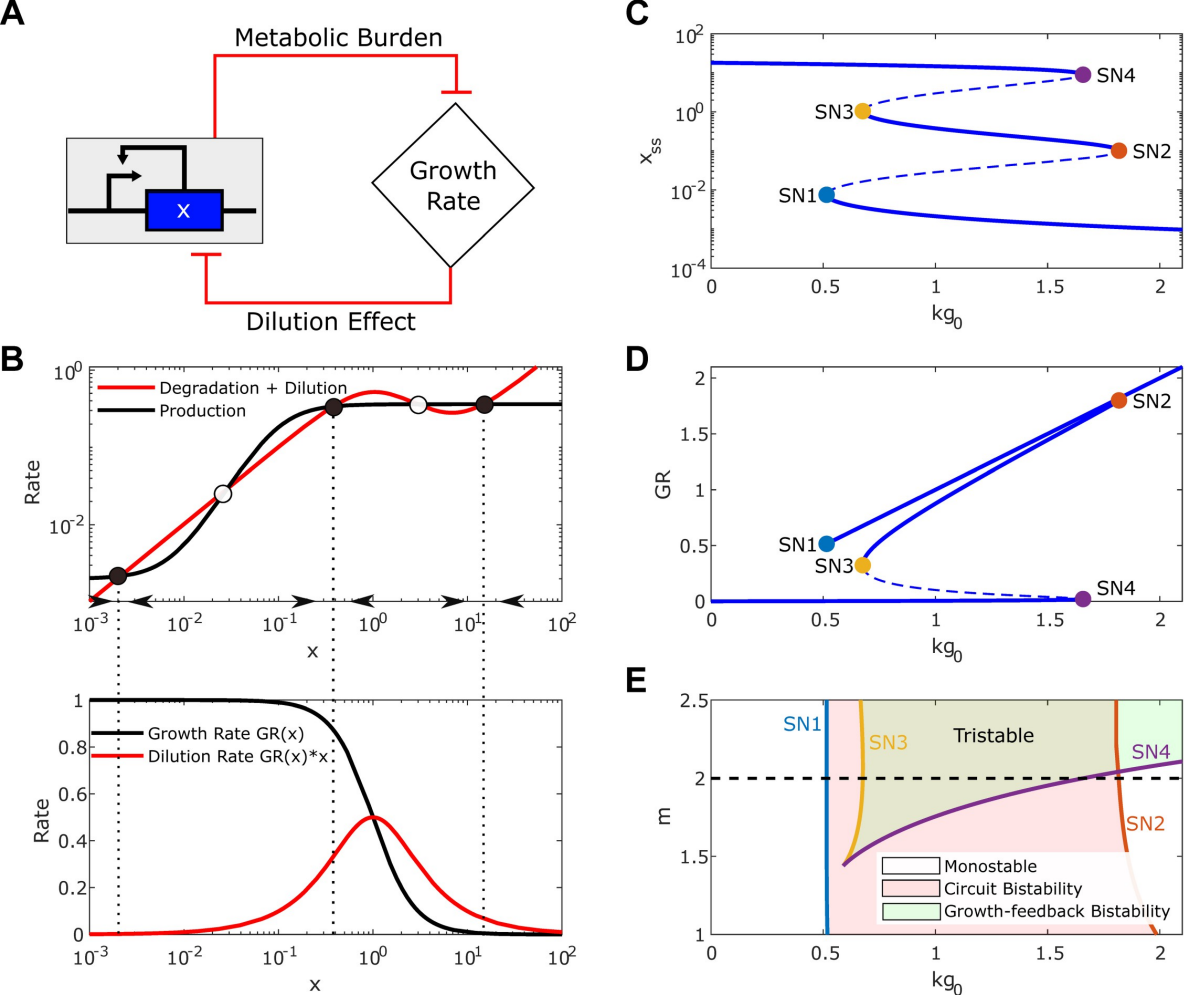
Tristability Emergence in the self-activation switch under ultrasensitive growth feedback. (A) Mutual inhibition interaction between growth rate and self-activation circuit. (B) The rate balance plot shows the emergence of tristability (top) based on the production rate curve (*f*(*x*), black line) and degradation plus dilution rate *d*(*x*) (red line, top) versus gene expression *x*. Solid and open circles represent stable and unstable steady states. The black arrows denote the directional field. The corresponding growth rate (black line) and dilution rate (red line) curves are shown in the bottom panel. Vertical dotted lines between the top and bottom panels represent the x values at stable steady state. (C) Bifurcation diagram of the gene expression x with respect to *kg*_0_. Solid and dashed lines correspond to the stable and unstable steady states. (D) Bifurcation diagram of growth rate with respect to *kg*_0_. The solid circles in C-D mark Saddle-Nodes SN1~4. (E) Two-parameters bifurcation diagram with respect to m and *kg*_0_ shows the dependence of the Saddle-Nodes SN1 (blue line), SN2 (orange line), SN3 (yellow line), and SN4 (purple line) on *m*. The region encloses between SN1 and SN2 corresponds to the bistability from the self-activation circuit. The region between SN3 and SN4 represents the bistability from ultrasensitive growth feedback. The overlap region of the two bistable regions gives tristability.

We further extend the above analysis to a general self-activation gene circuit (**Fig 2A**). Applying the general modeling framework to a self-activation circuit gives the following model,

$$\frac{dx}{dt}=k_{0}+k_{1}*\frac{x^{n}}{x^{n}+K^{n}}-d_{0}*x-GR(x)*x;$$

$$GR(x)=\frac{kg_{0}}{{\left(x/J\right)}^{m}+1},$$

Here, the production rate is modeled with $f(x)=k_{0}+k_{1}*\frac{x^{n}}{x^{n}+K^{n}}$, where *k*_0_ is the basal production rate, *k*_1_ the maximum production rate, *K* the dissociation constant, and *n* the hill coefficient. For a cooperative self-activation circuit, the *f*(*x*) is ultrasensitive as a function of x, shown as a sigmoid curve in **Fig 2B** (black curve, *n* = 2).

With a strong self-activation, the system could be bistable without considering the metabolic burden. Three steady-states can be found at the intersections between the production curve and the degradation curve. However, when the metabolic burden is considered, the degradation *d*(*x*) curve bends down and could intersect the function *f*(*x*) curve up to five times (**Fig 2B,** *m* = 2). Thus, the system can have five steady-states; three are stable, and two are unstable. The three stable steady-states are low gene expression (OFF-state) with a high growth rate, moderate gene expression (ON-state) with a slightly reduced growth rate, and high gene expression (super ON-state) with a low growth rate and dilution rate (**Fig 2B**). Hence, a bistable system from a cooperative self-activation circuit becomes tristable under high-order growth feedback.

The bifurcation diagram shows the steady-state values of the gene expression (*x*_*ss*_) and growth rate (*GR*) as a function of the maximum growth rate *kg*_0_ **(Fig 2C–2D)**. It is noted that four saddle-node points (SN1~4) were found, indicating the emergence of more stable qualitative states in the system. The three stable states are shown as solid branches. The first switch (indicated by SN1 and SN2) shows a similar growth rate (*GR*) but a considerable difference in gene expression (*x*) (**Fig 2C–2D**), which results from the positive feedback loop of the SA gene circuit. The second switch (indicated by SN3 and SN4) displays a significant difference in growth rate and gene expression produced by the growth feedback loop. For *kg*_0_ values lower than the smallest SN point (SN1 solid blue circle) or higher than the largest SN point (SN4, solid purple circle), the system is monostable with a high expressed gene and a low growth rate or a low expression gene expression and a high growth rate, respectively. Between the two left SN points (SN1<*kg*_0_<SN3) or two right SN points (SN2<*kg*_0_<SN4), the system is bistable with two of the three states. Interesting, the coexistence of all the three states (i.e., tristability) is found between two middle SN points (SN3<*kg*_0_<SN4).

We further performed a two-parameter bifurcation analysis with respect to *kg*_0_ and *m*. **Fig 2E** illustrates the positions of the saddle-node points with respect to *kg*_0_ by varying the *m*. The *m*−*kg*_0_ space is divided into five regions. The white region represents the system in the monostable OFF state for low values of *kg*_0_, or monostable ON state for low values of m and high values of *kg*_0_, respectively. In the red region, the system is bistable, which results from the gene circuit, while in the green region, the system is bistable but from growth feedback. The overlapped region with both red and green is tristable. It is worthy to note that the tristability area decreases as the SN3 (yellow line) and SN4 (purple line) get closer until merging with decreasing values of *m*, similar to the system with a constitutive promoter (**Fig 1E**). Therefore, reducing the values of *m* makes the system lose one switch mediated by growth feedback. Contrastingly, SN1 (blue line) and SN2 (orange line) exist for all values of *m* between 1 and 2. These results suggest that the bistability enclosed of SN3 and SN4 is determined by the high-order growth feedback (**Fig 2E** green region), and the bistability marked by SN1 and SN2 is governed by the cooperative self-activation circuit (**Fig 2E** red region). In summary, the underlying mechanism of tristability lies in the coupled cooperative self-activation feedback in the circuit and the growth feedback between the circuit and the host cell.

### Tristability emergence or enhancement of bistability depends on the synergism between the circuit feedback and growth feedback

To further understand the synergy between the circuit feedback and growth feedback on circuit dynamics, we performed a bifurcation analysis with varying parameter values in the self-activation circuits. We choose two key parameters that affect the self-activation circuit’s bistability capacity, the hills coefficient *n* representing the sensitivity of the circuit feedback, and *K* representing the threshold of the self-activation.

**Fig 3:**
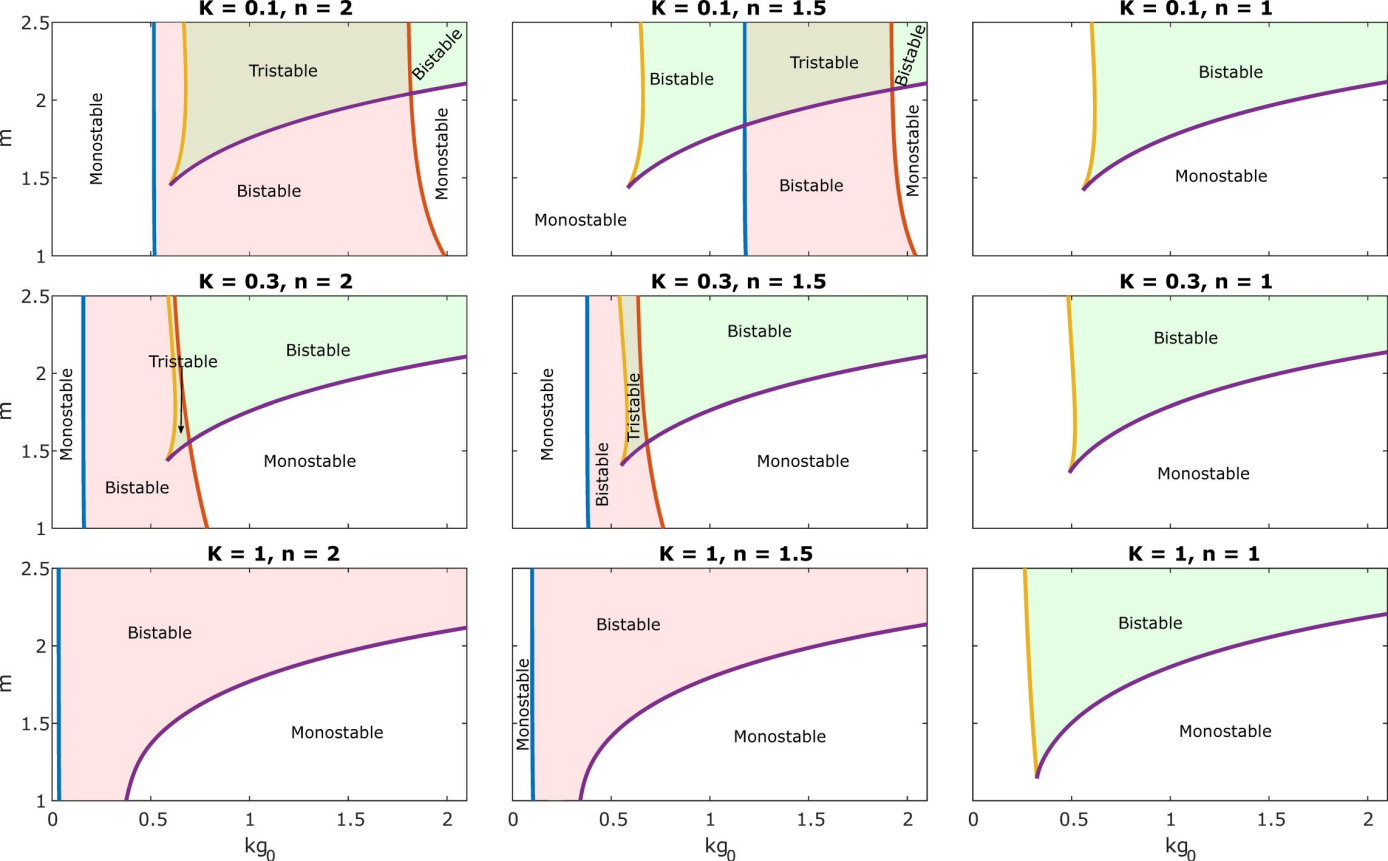
Tristability emerges from synergism between the self-activation circuit feedback and growth feedback. Two-parameter bifurcation diagram with respect *m* and *kg*_0_, for different values of hills coefficient n representing the sensitivity of the circuit feedback and *K* representing the threshold of the self-activation. Solid blue, orange, yellow, and purple lines represent saddle-node SN1~SN4, respectively.

**Fig 3** shows the two-parameters bifurcation with respect to *kg*_0_ and *m*, for different values of *n* and *K*. It is noted that with *K* fixed at 0.1, decreasing the value of *n* shifts the SN1 and SN2 curves right until they disappear at *n* = 1 where the system loses the circuit bistability (first rows in **Fig 3** and first/second rows in **S2 Fig**). In contrast, the SN3 and SN4 curves do not move with changes in *n* values indicating that the growth bistability is unaffected. On the other hand, with fixed *n* at 2 and *K* at 0.1, varying m value does not change SN1 and SN2 curves significantly but could collapse SN3 and SN4, leading to the loss of growth bistability (first two panels in the first row of **Fig 3,** and first/second columns in **S2 Fig**). This analysis reveals that the two switches are independently regulated by changing the circuit sensitivity and growth feedback sensitivity.

However, the interdependence between circuit and growth bistability differed with varying *K* values. SN3 and SN2 were lost with an increase of *K* values, representing the loss of the middle branch, which belongs to both circuit and growth switches (**Fig 3** first/second columns**, S3 Fig** first/second rows). That is, the system loses tristability, but the two feedback are now synergistic to make the system bistable. Decrease of *m* shrinks the bistable range of this synergetic switch (second/third rows in **Fig 3,** second/third columns in **S3 Fig**). Thus, growth feedback could enhance the robustness of the circuit bistability in the region where tristability was lost. Taken together, the emergence of the tristability or enhancement of bistability depends on the synergism between the circuit feedback and growth feedback.

### Tristability appears in the synthetic toggle switch circuit under ultrasensitive growth feedback

**Fig 4:**
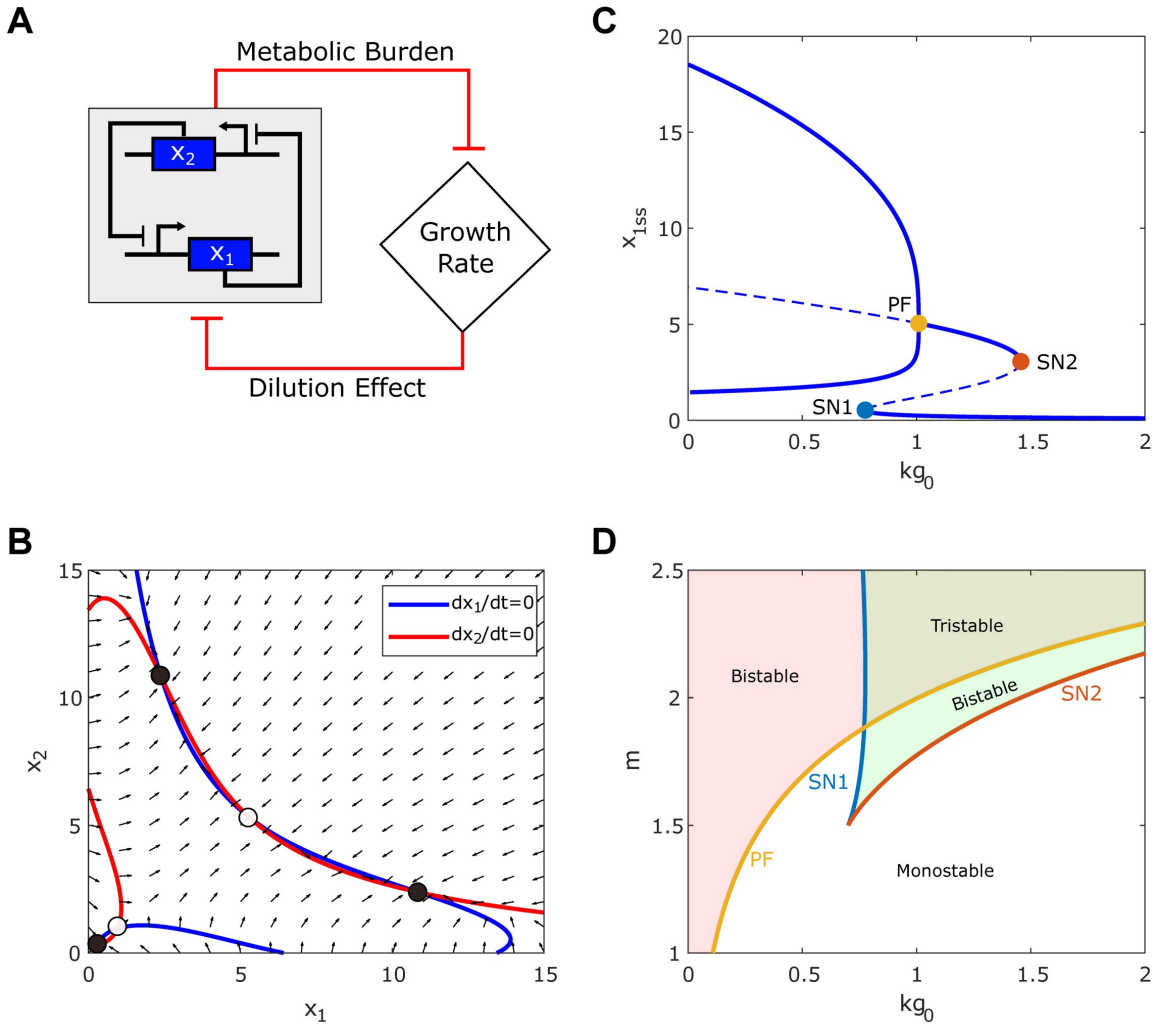
Tristability appears in the synthetic toggle switch circuit under the ultrasensitive growth feedback. (A) Diagram of interaction between the toggle switch circuit and growth rate. (B) Nullcline analysis shows the emergence of tristability. Red and blue lines represent the nullcline (*dx*_1_/*dt* = 0, *dx*_2_/*dt* = 0) for *x*_1_ and *x*_2_, respectively. Solid and open circles represent stable and unstable steady states. Black arrows represent the directional field of the system. (C) The bifurcation diagram of *x*_1_ with respect to *kg*_0_. Solid and dashed lines correspond to stable and unstable steady state points. Solid circles denote the saddle-nodes (blue and orange for SN1-2, respectively) and pitchfork bifurcation point (yellow for PF). (D) Two-parameter bifurcation diagram with respect to *m* and *kg*_0_ shows the dependence of the saddle-nodes (SN1-2) and pitchfork bifurcation point (PF) on *m*. SN1 (blue line) and SN2 (orange line) correspond to the saddle-node bifurcation points. Yellow line represents the PF point. The left red region of the blue (SN1) and orange (SN2) lines represents the bistability of the toggle switch circuit. The green region enclosed between the orange lines corresponds to the system bistability due to the growth feedback. Tristability is found where both bistable regions intersect.

Our previous work showed how circuits interact with growth feedback depending on their network topologies [21,22]. Here, we study how the qualitative state of the toggle switch circuit is affected under the ultrasensitive growth feedback. The toggle switch circuit consists of mutual inhibition of two genes, represented by *x*_1_ and *x*_2_ (**Fig 4A**). Applying the general modeling framework to a toggle switch circuit, we built the following model,

$$\frac{dx_{1}}{dt}=k_{0,1}+k_{1,1}*\frac{K_{1}^{n_{1}}}{x_{2}^{n_{1}}+K_{1}^{n_{1}}}-d_{0,1}*x_{1}-GR(x_{1},x_{2})*x_{1};$$

$$\frac{dx_{2}}{dt}=k_{0,2}+k_{1,2}*\frac{K_{2}^{n_{2}}}{x_{1}^{n_{2}}+K_{2}^{n_{2}}}-d_{0,2}*x_{2}-GR(x_{1},x_{2})*x_{2};$$

$$GR(x_{1},x_{2})=\frac{kg_{0}}{{\left(\frac{x_{1}+x_{2}}{J}\right)}^{m}+1},$$

where *k*_0,*i*_ is the basal expression rate for gene-*i*, *k*_1,*i*_ is the maximum inducible expression rate of gene-*i*, *K*_*i*_ is the concentration of its repressor producing half inhibition of the inducable expression rate of gene-*i*, *n*_*i*_ is the repression sensitivity of gene-*i*.

**Fig 4B** shows the nullclines (blue and red curve) and direction field (black arrows) for the toggle switch under ultrasensitive growth feedback (*m* = 2). Each intersection between the nullcline represents a steady-state (circles). Notice that there are five steady states, of which three are stable (solid circle), and two are unstable (non-solid circle). The stable steady states are $\left(x_{1}^{High},x_{2}^{Low}\right)$ or $\left(x_{1}^{Low},x_{2}^{High}\right)$ with a low growth rate and $\left(x_{1}^{Low},x_{2}^{Low}\right)$ with a high growth rate. Hence, tristability could also appear in a toggle switch circuit under ultrasensitive growth feedback.

**Fig 4C** shows the bifurcation diagram with respect to the *kg*_0_ (*m* = 2), where solid and dash lines symbolize a stable and unstable point, respectively. Our analysis reveals the existence of three bifurcations points (solid circle **Fig 4C**), two saddle-node (SN) points, and one pitchfork bifurcation (PF) point. For low values of *kg*_0_ (<SN1), the system shows the native bistability, which results from the double-negative feedback in the circuit. However, increasing of *kg*_0_ value enhances the dilution of circuit gene expression and weakens the feedback strength of the circuit. The native bistability from the circuit disappears at the PF point, above which the two stable states merge into one state with two genes coexpressed at moderate levels. SN1 and SN2 mark the switch that results from the growth feedback, similar to the constitutively expressing circuit (**Fig 1D**) and the self-activation circuit (**Fig 2C**). Notice that the tristability occurs for values of *kg*_0_ between SN1 and PF.

We performed additional bifurcation analysis with various combinations of Hill coefficients in the toggle switch and growth feedback. As shown in S4 Fig, we found that increasing the value of the metabolic sensitivity (*m*) not only gives a larger bistable range for the toggle switch but also enhances the chances of tristability. With *m* = 1, we only have a narrow range of *kg_0_* for the system to be bistable and no tristability, but the increase of *m* to 3 significantly increases the bistable range and shows the emerged tristability. On the other hand, with an increase of the Hill coefficient for toggle switch (*n*) value, both the bistable and tristability ranges increase. **Fig 4D** shows the two-parameter bifurcation with respect to *kg*_0_ and *m*, which reveals that the saddle nodes merge for decreasing value of *m*, consistent with the constitutively expressing circuit (**Fig 1E**) and the self-activation circuit (**Fig 2E**). On the other hand, the *kg*_0_ value corresponding to the PF point increases with *m*, resulting in an increased bistable region and the emergence of a tristability region in the top-right corner. Thus, our analysis suggests that tristability could result from a synergy between the growth feedback and the toggle switch.

The existence of pitchfork bifurcation requires a perfectly symmetric parameter setting. An asymmetric parameter setting with *k*_1,1_ ≠ *k*_1,2_ indeed makes the pitchfork bifurcation disappears. However, another saddle-node bifurcation (SN3) emerges, and tristability can also be seen for a range of *kg*_0_ (**S5A Fig**). The bifurcation diagram in terms of the production rate of gene *x*_1_ (*k*_1,1_) (**S5B Fig**) shows a stepwise ternary switch, which also confirms the existence of tristability with *k*_1,1_ values different from *k*_1,2_. Hence, the emergent tristability does not require a symmetrical parameter setting.

**Fig 5:**
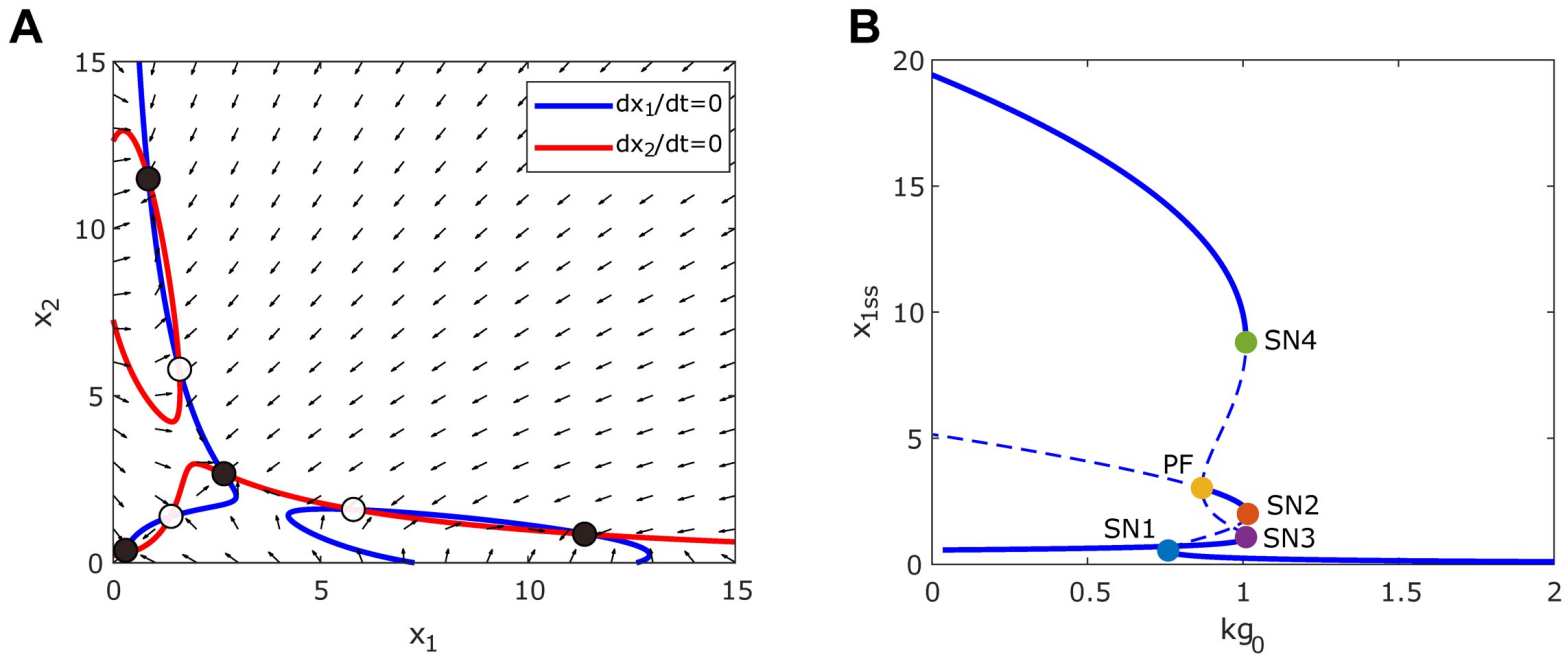
Quadstability emerged in the toggle switch circuit under ultrasensitive growth feedback. (A) Nullcline analysis shows the emergence of quadstability. Red and blue lines represent the nullcline for *x*_1_ and *x*_2_, respectively. Solid and non-solid circles correspond to stable and unstable steady states. Black arrows represent the directional field. (B) The bifurcation diagram of *x*_1_ with respect *kg*_0_. The solid circles reveal four saddle-nodes (blue, orange, purple, and green for SN1~4) and pitchfork (yellow, PF) bifurcation points.

We further tuned the circuit parameters to see whether there is a topology dependence effect under ultrasensitive growth feedback. We found that with lower mutual inhibition thresholds (*K*_1_ and *K*_2_ values), the system could have four stable steady states, which is different from the case of self-activation. **Fig 5A** shows the nullclines and directed field of this scenario, where seven steady states were found, four of which are stable, and three are unstable. While three of the stable states are the same as in the case of tristability (**Fig 4A**), one additional state with moderate coexpression of both genes emerges. That is, the toggle switch circuit could show quadstability under ultrasensitive growth feedback. Furthermore, we performed a one-parameter bifurcation with respect to *kg*_0_ to determine the mechanism of quadstability (**Fig 5B**). Our analysis reveals the existence of two additional saddle nodes (SN3 and SN4), which squeeze the coexpressing state within the original tristable region. Therefore, a toggle switch circuit with a relatively strong mutual inhibition could be quadstable under ultrasensitive growth feedback.

## Discussion

Our results suggest that the growth feedback could alter the number of qualitative steady states in a host-circuit system. Additional qualitative states could be found on gene circuits with various topologies, including constitutive promoter, self-activation, and toggle switch, under ultrasensitive growth feedback. The underlying mechanism is that the changes in gene expression coincide with a metabolic and physiological adaptation of the host cell, which could lead to some cells expressing a high level of an extrageneous gene with a slow growth rate and some cells expressing lower levels with a fast growth rate. The emergence of qualitative states also lies in the coordinated coupling of growth feedback and circuit feedback as well as circuit topology. While the self-activation circuit could be tristable with one additional state, the toggle switch circuit could be tristable or quadstable with two additional states. Recently, Ye et al. showed that quadstability could be achieved by coupling three feedback loops in the system [26]. Our study provides another possibility where quadstability could emerge with two ultrasensitive positive feedback loops.

Here, we investigate the mechanism of the potential emergence of the qualitative state in synthetic gene circuits due to growth feedback. However, we do not fully understand the underlying molecular mechanism. The values of m could be related to many factors, including nutrient quality, host strain, gene characteristics, and the molecular regulation of the metabolic control in the host cell. The molecular and cellular organization of the host system for metabolic regulation, such as the growth regulation by ppGpp, could be one of the essential mechanisms. Future experiments would be needed to understand the mechanism that defines the nonlinear growth rate and gene expression relation and the dependence of the metabolic burden sensitivity and related physiological characteristics on the nutrient level in the culture media and bacterial strain types. This will inform us of the strategies for engineering the host cells for tunable ultrasensitivity and exploiting these additional qualitative states for generating novel dynamics and functions. To experimentally observe these states, we need to keep the growth heterogeneity among the cell populations in mind as slow-growing cells are outgrown by the fast-growing ones [24,25]. Thus, it is necessary to investigate this at the single-cell level using the ‘Mother Machine’ [27–29], in which the new daughter cell flows out so that the slow-growing population can be best reserved.

For simplicity, here we did not consider the effect of growth rate on the production rate of the synthetic gene. The growth rate has global effects on gene expression, especially the transcription rate [16,18]. In addition, the dependence of one specific gene on growth rate could depend on how it is regulated. For example, if it is nutrient-modulated, which leads to negative regulation, or if it is translation-modulated, which leads to positive regulation [18]. Recently, we used a coarse-grained model to understand the mechanism of emergent damped oscillation induced by nutrient-modulating growth feedback [22]. Data fitting and theoretical analysis suggest a nonmonotonic growth-rate regulation of gene production rate [22]. This gene or circuit-specific effects still need to be determined experimentally. The dilution effect mediated by host cell growth is more general to be considered here. Future works need to examine the circumstances under which the exogenous gene production is influenced by the growth rate. The growth could have a dual role on exogenous gene expression under these conditions and make the circuit-host system even more complicated with multiple feedback loops mediated by host cell growth, leading to the addition or loss of states.

Here our analysis was based on the assumption of maintaining cells at the exponential stage. Growth condition shift could also alter the steady-state of the circuit-hot system. For example, we previously found that the self-activation circuit lost its memory after diluting the cell into fresh media [21]. Thus, memory loss or state emergence depends on the cellular context. In addition, it was reported that nutrient shift also changes the physiological state of the host cell [13,30,31]. Thus, it will be interesting to systematically analyze the switching behavior between these potential states upon nutrient shift, which will help understand the mechanism of switch reversibility. Here we focused on the gene-growth feedback in the context of synthetic biology, but the natural systems also use it. For example, cell-cycle-mediated feedback was used to control myeloid differentiation [32]. The horizontal gene transfer during evolution could cause similar growth feedback and leads to the emergence of new states into the natural system.

## Methods

### a. Model parameter values

Here we focused on the general conclusion, which does not depend on the specific parameter setting but relies more on their relative values. The range of values for maximum growth rate *kg*_0_ is set from 0~2/*h*, which is obtained from published experimental data shown in Fig 1B [14]. The key parameter for the emergence of the extra stable states depends on the parameter condition $\frac{kg_{0}}{d_{0}}>\frac{4m}{{\left(m-1\right)}^{2}}$ (see Note A in S1 Text for more details). The half-lives of GFP variants in *E*.*Coli* has a very broad range from 40 minutes to 26 hours, which gives us the degradation rate constant (*d*_0_) from 0.012~0.45/*h* [33,34]. This gives a broad reasonable range for these parameters. For demonstration, the parameter values used in the figures are as follows. For gene circuit with a constitutive promoter: *k*_0_ = 0.1 *a*.*u*./*h*, *d*_0_ = 0.0015/*h*, *J* = 1 *a*.*u*., and *kg*_0_ = 0.9/*h* for Figs 1C and S1C–S1D. For gene circuit with self-activation: *k*_0_ = 0.002 *a*.*u*./*time*, *k*_1_ = 0.36 *a*.*u*./*h*, *K* = 0.1 *a*.*u*., *n* = 2, *d*_0_ = 0.02/*h*, *J* = 1 *a*.*u*., and *kg*_0_ = 1/*h* for Fig 2B. For the toggle switch circuit, tristable case in Fig 4B: *k*_0,*i*_ = 0.001 *a*.*u*./*h*, *k*_1,*i*_ = 0.2 *a*.*u*./*h*, *K*_*i*_ = 5 *a*.*u*., *n*_*i*_ = 2, *d*_0,*i*_ = 0.01/*h*, *J* = 1 *a*.*u*., and *kg*_0_ = 0.9/*h*. For the quadstable case in Fig 5A: *K*_*i*_ = 3 *a*.*u*., and *kg*_0_ = 0.95/*h*.

### b. Parameter estimation for the qualitative relation between growth rate and exogenous gene expression

To estimate the sensitivity of metabolic burden, we used the function $GR=\frac{kg_{0}}{{\left(x/J\right)}^{m}+1}$ to fit the experimental data of growth rate versus exogenous gene portion quantified from Ref. [14]. Using the Curve Fitter app (https://www.mathworks.com/products/curvefitting.html) on MATLAB R2021b, we found the corresponding value of *m* in three different culture mediums for *E*.*coli*, 1.456, 1.76, and 1.424 (See Fig 1B), respectively, indicating high-sensitive growth feedback.

### c. Bifurcation analysis

In order to study the qualitative steady-state behaviors of the system, bifurcation analysis was performed using the software "oscill8" (http://oscill8.sourceforge.net/) and "MatCont" (https://sourceforge.net/projects/matcont/) on MatLab R2021b [35].

## Supporting information

S1 Fig Analysis of the parameter condition for the bistability emergence in the simple gene circuit with a constitutive promoter. (A) Parameter condition for the existence of local maximum and minimum for degradation plus dilution rate versus gene expression (*d*(*x*)) in the space of *m* and *kg*_0_. Colormap shows the difference between local maxima and local minima (Δ*d* = *d*(*x*_*max*_)−*d*(*x*_*min*_))). (B) The degradation plus dilution rate *d*(*x*) (solid line) over gene expression (x) with various combinations of *m* and *kg*_0_ as shown in A. The dotted and dash-dotted lines represent the curves of degradation *d*0*x and degradation plus max dilution (*d*_0_*x+*kg*_0_*x), respectively. (C) The bifurcation diagram of gene expression with respect to *k*_0_. Solid and dash lines represent stable and unstable steady states, respectively. Solid circles mark saddle-node bifurcation points (SN1~2). (D) Two-parameter bifurcation of gene expression with respect to *m* and *kg*_0_ shows the dependence of the saddle-nodes (SN1~2) on *m*.
(TIF)

S2 Fig Bifurcation diagrams of the steady-state gene expression in the self-activation circuit with respect to *kg*_0_ for different combinations of *m* and *n*. Solid and dashed lines correspond to the stable and unstable steady states. The solid circle marks saddle-nodes (SN1~4).
(TIF)

S3 Fig Bifurcation diagrams of the steady-state gene expression in the self-activation circuit with respect to *kg*_0_ for different combinations of *m* and *K*. Solid and dashed lines correspond to the stable and unstable steady states. The solid circle marks saddle-nodes (SN1~4).
(TIF)

S4 Fig The bifurcation diagram of the steady-state gene expression in the toggle switch circuit with respect to *kg*_0_, for combination of *m* and *n*. Solid and dashed correspond to stable and unstable steady states. The solid circles denote the saddle-nodes (SN1-2, blue/orange dots) or pitchfork bifurcation points (PF, yellow dots).
(TIF)

S5 Fig Bifurcation analysis for toggle switch circuit with the asymmetrical parameter setting. Solid and dashed lines represent stable and unstable steady states of *x*_1_, respectively. Dots represent the saddle-nodes (SN1~3). (A) Bifurcation diagram of *x*_1_ with respect to *kg*_0_, with *k*_1,1_ = 2.1. (B) Bifurcation diagram of *x*_1_ with respect to *k*_1,1_ with *kg*_0_ = 0.8. Vertical dash-dotted line represents the values of *k*_1,1_ = *k*_1,2_.
(TIF)

S1 Text **Note A. Condition for the existence of bistability in the simple gene circuit with a constitutive promoter. Note B. Nondimensionalizing the mathematical models**.
(PDF)
