# Supplementary figures and images for: Emergence of qualitative states in synthetic circuits driven by ultrasensitive growth feedback
Source: PLoS Comput Biol. 2022 Sep 16;18(9):e1010518. doi: 10.1371/journal.pcbi.1010518 (PMC9518899; doi:10.1371/journal.pcbi.1010518)

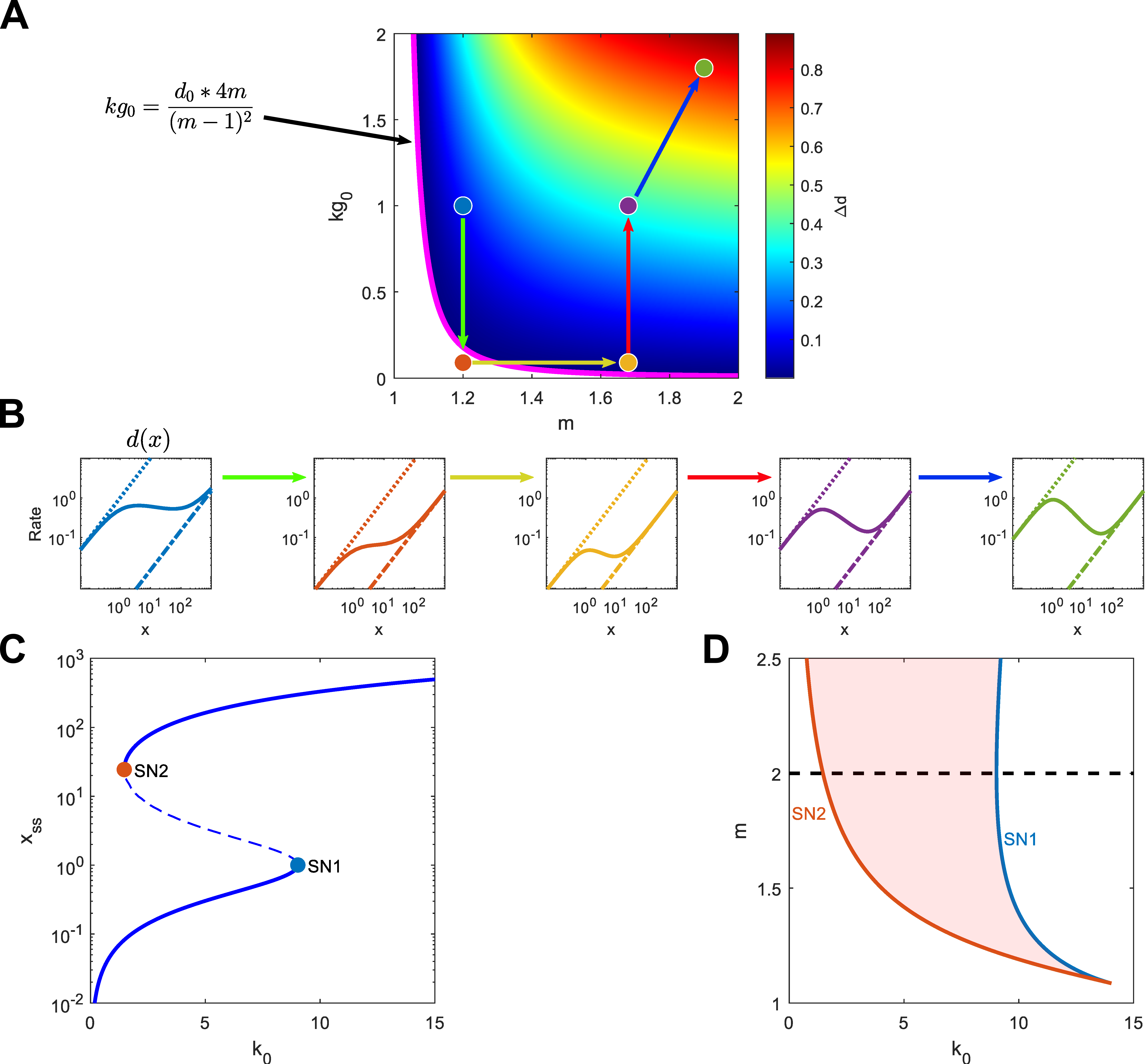

Supplement: S1 Fig — (A) Parameter condition for the existence of local maximum and minimum for degradation plus dilution rate versus gene expression (d(x)) in the space of m and kg0. Colormap shows the difference between local maxima and local minima (Δd = d(xmax)−d(xmin))). (B) The degradation plus dilution rate d(x) (solid line) over gene expression (x) with various combinations of m and kg0 as shown in A. The dotted and dash-dotted lines represent the curves of degradation d0*x and degradation plus max dilution (d0*x+kg0*x), respectively. (C) The bifurcation diagram of gene expression with respect to k0. Solid and dash lines represent stable and unstable steady states, respectively. Solid circles mark saddle-node bifurcation points (SN1~2). (D) Two-parameter bifurcation of gene expression with respect to m and kg0 shows the dependence of the saddle-nodes (SN1~2) on m. (TIF) [file pcbi.1010518.s001.tif]

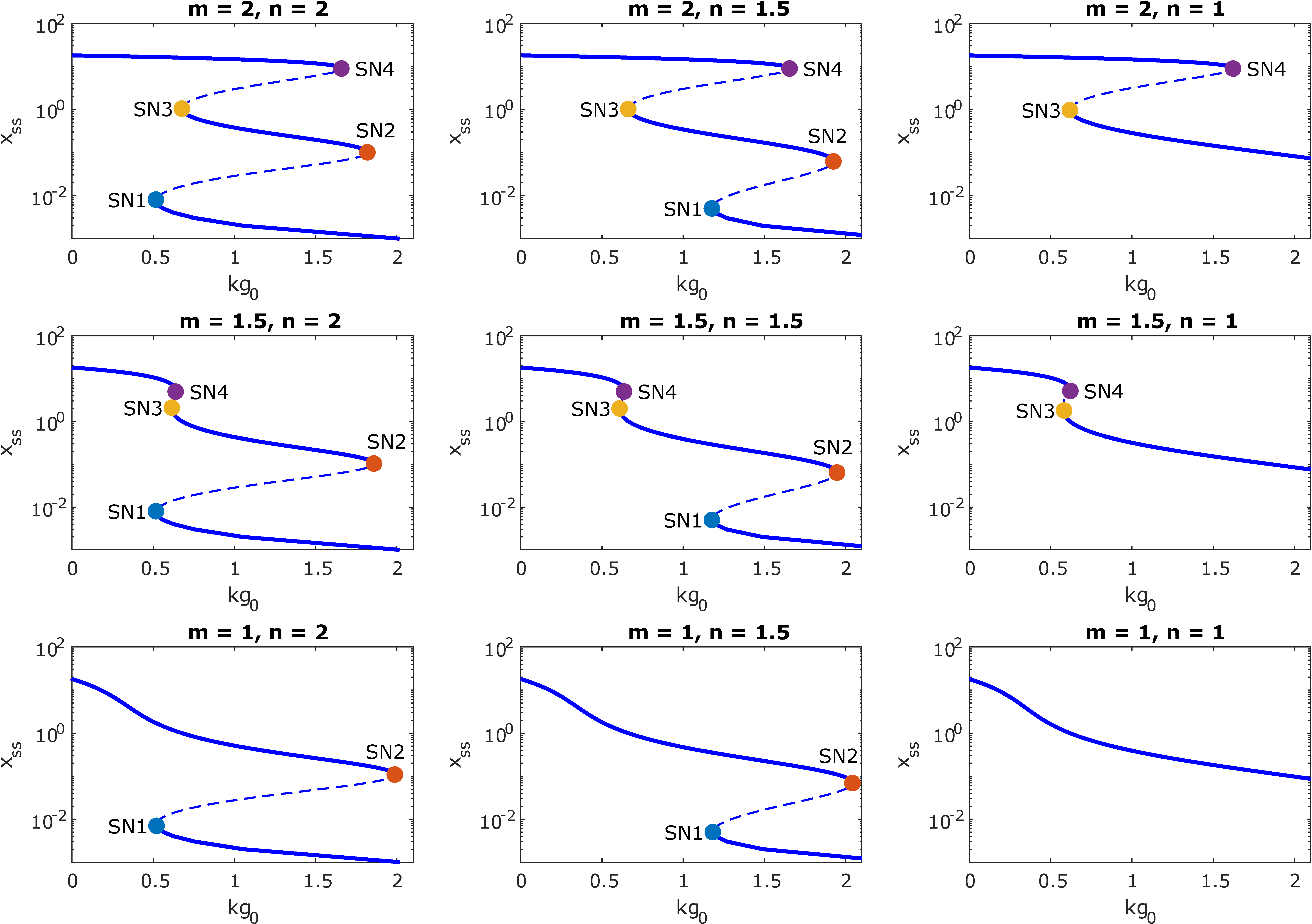

Supplement: S2 Fig — Solid and dashed lines correspond to the stable and unstable steady states. The solid circle marks saddle-nodes (SN1~4). (TIF) [file pcbi.1010518.s002.tif]

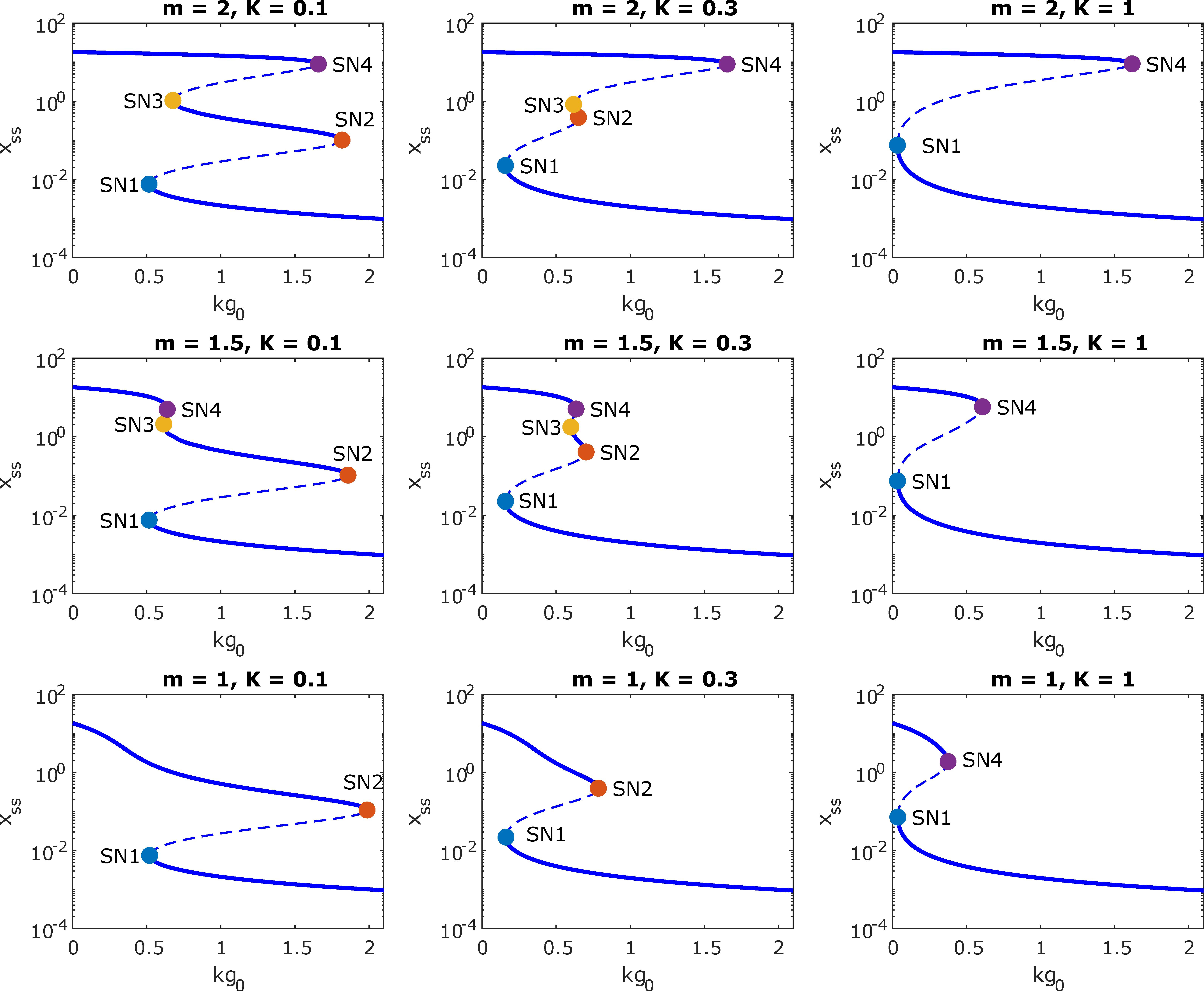

Supplement: S3 Fig — Solid and dashed lines correspond to the stable and unstable steady states. The solid circle marks saddle-nodes (SN1~4). (TIF) [file pcbi.1010518.s003.tif]

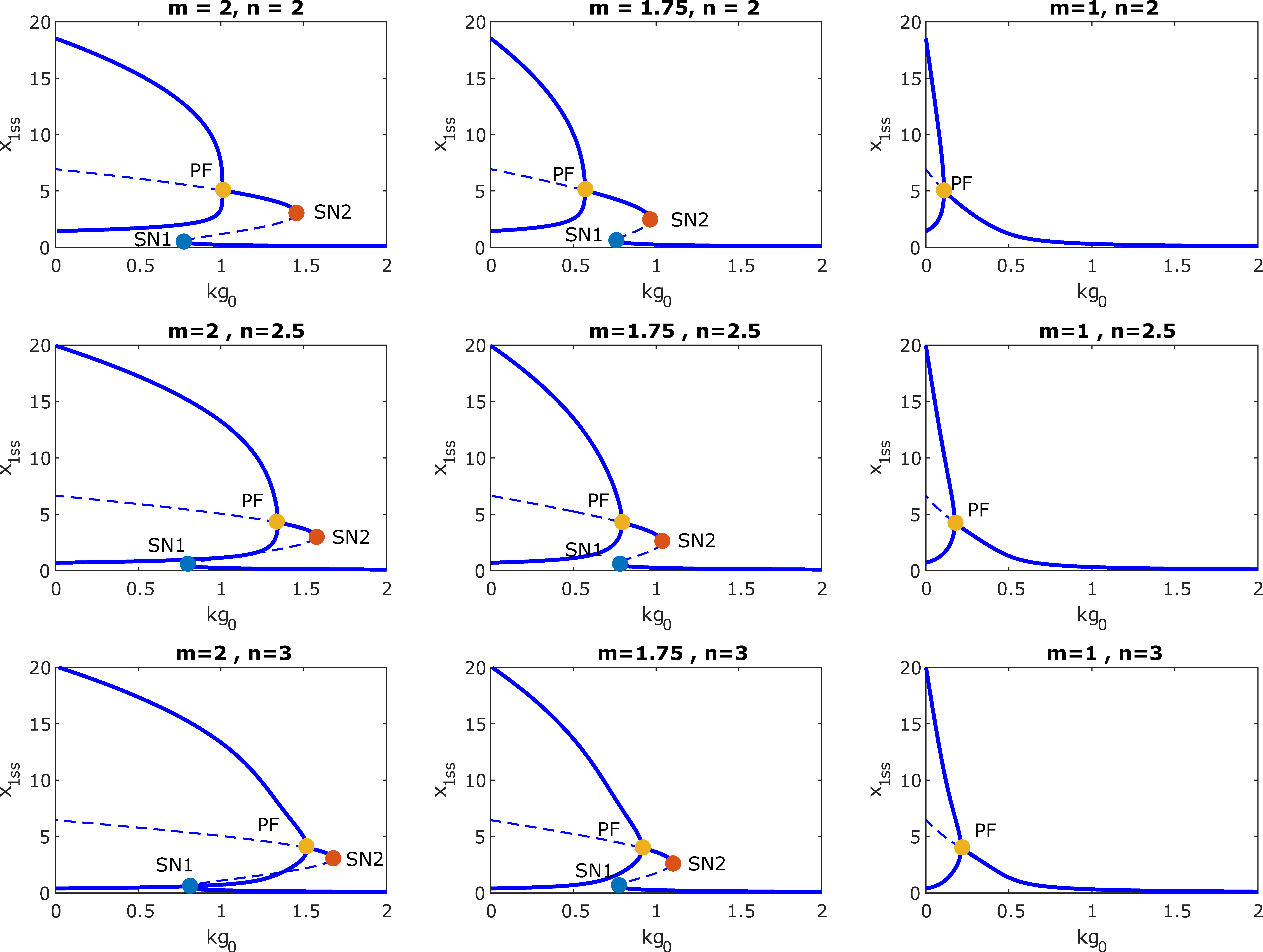

Supplement: S4 Fig — Solid and dashed correspond to stable and unstable steady states. The solid circles denote the saddle-nodes (SN1-2, blue/orange dots) or pitchfork bifurcation points (PF, yellow dots). (TIF) [file pcbi.1010518.s004.tif]

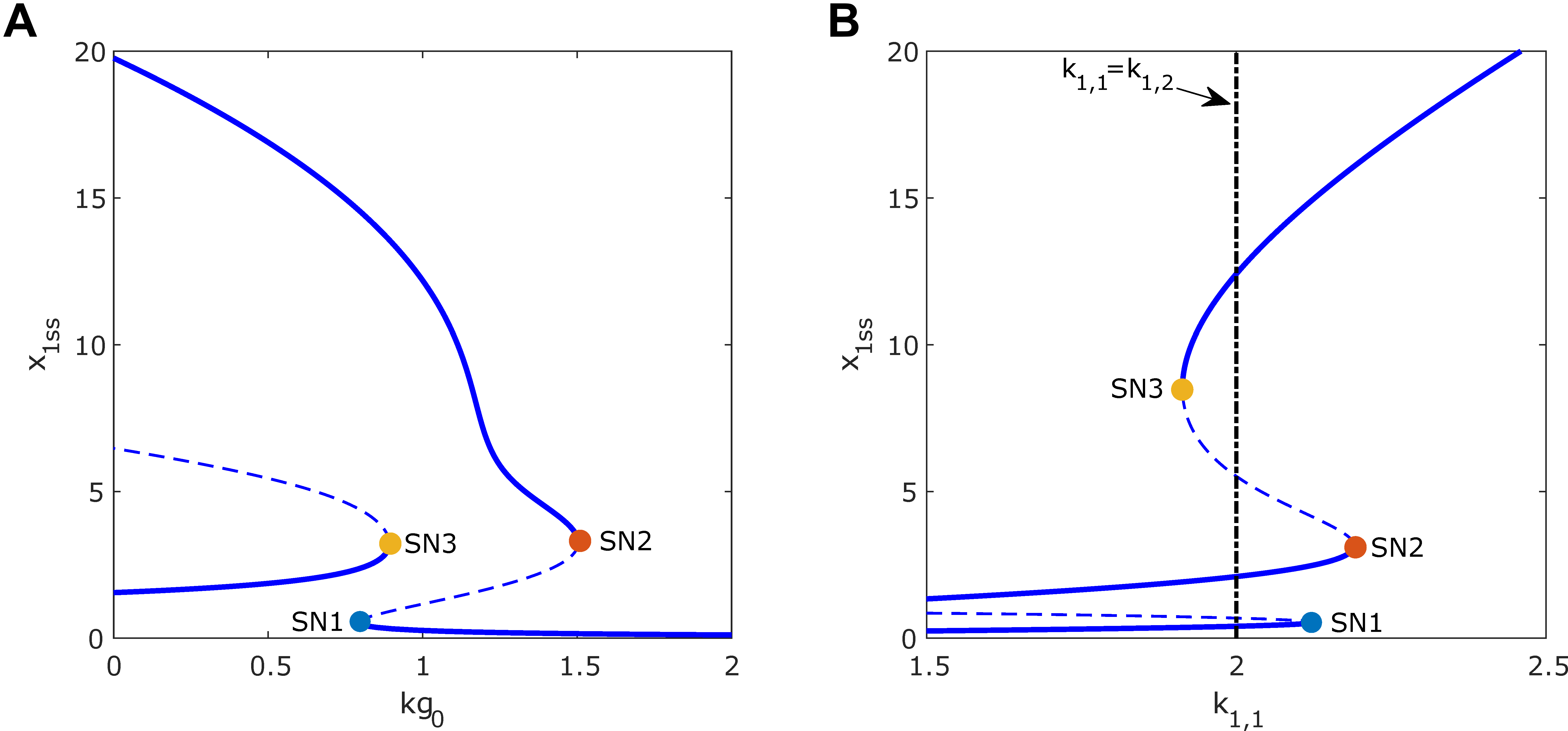

Supplement: S5 Fig — Solid and dashed lines represent stable and unstable steady states of x1, respectively. Dots represent the saddle-nodes (SN1~3). (A) Bifurcation diagram of x1 with respect to kg0, with k1,1 = 2.1. (B) Bifurcation diagram of x1 with respect to k1,1 with kg0 = 0.8. Vertical dash-dotted line represents the values of k1,1 = k1,2. (TIF) [file pcbi.1010518.s005.tif]
