# Supplementary material for: Emergence of qualitative states in synthetic circuits driven by ultrasensitive growth feedback
Source: PLoS Comput Biol. 2022 Sep 16;18(9):e1010518. doi: 10.1371/journal.pcbi.1010518 (PMC9518899; doi:10.1371/journal.pcbi.1010518)
Supplement: S1 Text — Note A. Condition for the existence of bistability in the simple gene circuit with a constitutive promoter. Note B. Nondimensionalizing the mathematical models. (PDF) [file pcbi.1010518.s006.pdf]

Supporting Information

S1 Text

Note A: Condition for the existence of bistability in the simple gene circuit with a constitutive promoter.

In order to understand how the growth feedback mediated by the ultrasensitive dilution effect can generate an extra steady state in the simple gene circuit with a constitutive promoter, we analyze the mathematical function of degradation plus dilution curve $d(x) = d_0 * x + kg_0 * \frac{x}{\left(\frac{x}{J}\right)^m + 1}$. The emergence of the bistability needs to meet two conditions: 1) the existence of both local maxima and minima in the curve; 2) the production rate locates between the maxima and minima. Therefore, we first need to find the parameters condition that allows the function $d(x)$ to have local maximum and minimum for non-negative real values of x.

Therefore, in order to locate the local maximum and minimum on $d(x)$, we solve the equation $d'(x) = 0$, for non-negative values of x.

$$d'(x) = d_0 + kg_0 * \frac{\left(\frac{x}{J}\right)^m + 1 - x * \frac{m \cdot x^{m-1}}{J^m}}{\left(\left(\frac{x}{J}\right)^m + 1\right)^2}$$

$$= d_0 + kg_0 * \frac{1 - (m-1)\frac{x^m}{J^m}}{\left(\left(\frac{x}{J}\right)^m + 1\right)^2}$$

For simplification, we used $u = \left(\frac{x}{J}\right)^m$. Therefore we get

$$d_0 + kg_0 * \frac{1 - (m-1)u}{(u+1)^2} = 0;$$

$$d_0 * (u+1)^2 + kg_0 * (1 - (m-1)u) = 0$$

$$d_0 u^2 + \big(2d_0 - kg_0(m-1)\big)u + (d_0 + kg_0) = 0$$

Solving the equation for u we get:

$$u = \frac{-\big(2d_0 - kg_0(m-1)\big) \pm \sqrt{\big(2d_0 - kg_0(m-1)\big)^2 - 4d_0(d_0 + kg_0)}}{2d_0}$$

Thus,

$$x = J\left(\frac{-\big(2d_0 - kg_0(m-1)\big) \pm \sqrt{\big(2d_0 - kg_0(m-1)\big)^2 - 4d_0(d_0 + kg_0)}}{2d_0}\right)^{\frac{1}{m}}$$

To get two different real non-negative roots, we first need to find the parameter condition that makes the discriminant greater or equal to zero,

$$\left(2d_0 - kg_0(m-1)\right)^2 - 4d_0(d_0 + kg_0) \geq 0.$$

A simplified version the first condition:

$$\frac{kg_0}{d_0} \geq \frac{4m}{(m-1)^2}$$

In addition, we also need to follow the condition:

$$-\left(2d_0 - kg_0(m-1)\right) \geq 0$$

This led to the second condition:

$$\frac{kg_0}{d_0} \geq \frac{2}{(m-1)}.$$

For m>1

$$\frac{kg_0}{d_0} \geq \frac{4m}{(m-1)^2} = \frac{2m}{(m-1)} * \frac{2}{(m-1)} > \frac{2}{(m-1)}$$

Thus, the first condition $\frac{kg_0}{d_0} \geq \frac{4m}{(m-1)^2}$ guarantees real non-negative roots. That is, this is the only condition that allows the $d(x)$ curve to have a local maximum and minimum for non-negative real values of x.

In addition, the curve of the production rate of the synthetic gene circuit $f(x)$ need to be located between the maximum and minimum so that it intersects the d(x) three times in order to have an extra steady state,

$$d(x_{max}) < f(x) < d(x_{min})$$

where

$$x_{max} = J\left(\frac{-\left(2d_0 - kg_0(m-1)\right) - \sqrt{\left(2d_0 - kg_0(m-1)\right)^2 - 4d_0(d_0 + kg_0)}}{2d_0}\right)^{\frac{1}{m}}$$

$$x_{min} = J\left(\frac{-\left(2d_0 - kg_0(m-1)\right) + \sqrt{\left(2d_0 - kg_0(m-1)\right)^2 - 4d_0(d_0 + kg_0)}}{2d_0}\right)^{\frac{1}{m}}$$

Note B: Nondimensionalizing the mathematical models

The General model is given by:

$$\frac{dx_i}{dt} = f_i(x) - d_{0_i} * x_i - GR(x) * x_i$$

$$GR(x) = \frac{kg_0}{(\Sigma x_i/J)^m + 1}$$

In order to non-dimensiolinze the model, we define

$$\xi_i = \frac{x_i}{x_0}, \quad \tau = \frac{t}{t_0}$$

Therefore:

$$\frac{dx_i}{dt} = \frac{x_0}{t_0} * \frac{d\xi_i}{d\tau}.$$

Substituting the dimensionalized model, we get:

$$\frac{d\xi_i}{d\tau} = \frac{t_0}{x_0} * \frac{dx_i}{dt} = f_i(x) * \frac{t_0}{x_0} - d_{0,i} * x_i * \frac{t_0}{x_0} - GR(x) * x_i * \frac{t_0}{x_0};$$

If we set $x_0 = J$ and $t_0 = \frac{1}{d_0}$ (assuming $d_{0_i} = d_0$), we have the following non-dimensionalized general model

$$\frac{d\xi_i}{d\tau} = \frac{f_i(\xi_i * J)}{d_0 * J} - \xi_i - \widetilde{GR}(\xi_i) * \xi_i$$

$$\widetilde{GR}(\xi_i) = \frac{kg_0/d_0}{(\Sigma \xi_i)^m + 1}$$

For the Gene Circuit with a constitutive promoter, we have the following non-dimensionalized model

$$\frac{d\xi}{d\tau} = \alpha - \xi - \widetilde{GR}(\xi) * \xi;$$

$$\widetilde{GR}(\xi) = \frac{kg_0/d_0}{(\xi)^m + 1}$$

Where $\alpha = \frac{k_0}{d_0 * J}$.

For the self-activation Gene Circuit, we have the following non-dimensionalized model

$$\frac{d\xi}{d\tau} = \alpha + \beta \frac{\xi^n}{\xi^n + \kappa^n} - \xi - \widetilde{GR}(\xi) * \xi;$$

$$\widetilde{GR}(\xi) = \frac{kg_0/d_0}{(\xi)^m + 1}$$

Where $\alpha = \frac{k_0}{d_0 * J}$, $\beta = \frac{k_1}{d_0 * J}$, $\kappa = K/J$.

For the toggle Switch Gene Circuit, we have the following non-dimensionalized model

$$\frac{d\xi_1}{d\tau} = \alpha_1 + \beta_1 \frac{\kappa_1^n}{\xi_2{}^n + \kappa_1^n} - \xi_1 - \widetilde{GR}(\xi) * \xi_1;$$

$$\frac{d\xi_2}{d\tau} = \alpha_2 + \beta_2 \frac{\kappa_2^n}{\xi_1^n + \kappa_2^n} - \xi_2 - \widetilde{GR}(\xi) * \xi_2;$$

$$\widetilde{GR}(\xi) = \frac{kg_0/d_0}{(\xi_1 + \xi_2)^m + 1}$$

Where $\alpha_i = \frac{k_{0_i}}{d_0 * J}$, $\beta_i = \frac{k_i}{d_0 * J}$, $\kappa_i = K_i/J$.
